# Underground coal mining reduces organic carbon stability and alters microbial metabolic limitation in biocrusts of an arid region in northern China

**DOI:** 10.3389/fmicb.2026.1798269

**Published:** 2026-06-23

**Authors:** Yun Guo, ShaoPeng Ma, Li Ma, Benhua Sun, ManYin Zhang

**Affiliations:** 1 Institute of Geological Hazards Prevention, Gansu Academy of Sciences, Lanzhou, China; 2 Institute of Geographical Sciences, Hebei Academy of Sciences, Hebei Technology Innovation Center for Geographic Information Application, Shijiazhuang, China; 3 Postdoctoral Scientific Research Station of Geography, Hebei Normal University, Shijiazhuang, China; 4 Key Laboratory of Plant Nutrition and the Agri-Environment in Northwest China, Ministry of Agriculture, Northwest A&F University, Yangling, China

**Keywords:** biocrusts, carbon use efficiency, coal mining disturbance gradients, extracellular enzymes, soil organic carbon stability

## Abstract

Underground coal mining disrupts surface stability, depletes soil organic carbon (SOC) stocks through persistent alterations in topography and hydrological regimes, and adversely affects soil microbial diversity and ecological functioning. Biological soil crusts (biocrusts) play critical roles in soil stabilization, SOC sequestration, and microbial nutrient transformations in coal-mining areas. However, the mechanisms by which different mining disturbance gradients regulate organic carbon composition and stability, microbial metabolic processes, and nutrient limitations within biocrusts remain poorly understood. In this study, we investigated biocrust organic carbon fractions and microbial resource characteristics across coal mining subsidence areas by applying an organic carbon stability index and ecoenzymatic stoichiometry model to evaluate the effects of mining disturbance gradients (unmined, active mining, and 1–2 year mined-out) and vegetation types (herbaceous and shrub) on biocrust properties and microbial resource limitation. Biocrust cover ranged from 26.4 to 40.8%, with the highest moss cover occurring in herbaceous vegetation in unmined areas, whereas cyanobacterial biocrusts were most abundant under shrub vegetation in unmined areas. Compared with unmined sites, active mining and 1–2 year mined-out areas exhibited significantly lower recalcitrant organic carbon (ROC), dissolved organic carbon (DOC), and microbial biomass carbon (MBC) in biocrusts. Active mining markedly reduced biocrust nutrient availability, organic carbon stability, microbial enzyme activities related to carbon (C), nitrogen (N), and phosphorus (P) acquisition, and microbial carbon use efficiency (CUE). Vector analysis of extracellular enzyme activities indicated that moss biocrusts were primarily P-limited. In contrast, cyanobacterial biocrusts were predominantly N-limited, with nutrient limitations most pronounced in active mining and 1–2 year mined-out areas. Moreover, microbial carbon limitation was significantly greater in the moss biocrusts than in the cyanobacterial biocrusts. Partial least squares path modeling further demonstrated that coal mining influences microbial CUE, primarily by altering metabolic limitations and organic carbon stability, with microbial carbon limitation exerting the strongest negative total effect on CUE. Overall, these findings elucidated the key mechanisms governing the stability of biocrust organic carbon and the microbial metabolic constraints under mining disturbance, providing a scientific basis for ecological protection and restoration in arid and semi-arid mining regions.

## 1 Introduction

Coal has played a pivotal role in China’s energy mix, with an average annual consumption of 5.026 billion tons over the past 5 years, representing 57.14% of the country’s total energy consumption (Sun et al., 2024). This highlights the crucial contribution of coal to China’s economic development. Previous studies have reported that coal accounts for 56.8% of China’s primary energy consumption and that more than 95% of this coal is extracted from underground mines (Wang et al., 2020). By 2020, China’s extensive coal mining industry led to approximately 2,000,000 hm^2^ of land subsidence, increasing at an annual rate of 70,000 hm^2^. Underground coal mining causes a range of environmental issues, including damage to agricultural land, soil deformation, surface cracking, and destruction of water resources, all of which severely affect plant roots and buried plant communities (Bi et al., 2019; Bai et al., 2022), ultimately disrupting plant growth and ecosystem stability. In addition, underground coal mining alters the SOC pool. Studies have shown that mining reduces soil carbon stocks by permanently altering topography and geological structures as well as by disrupting surface and subsurface hydrological regimes (Maharaj et al., 2007). Moreover, mining activities exacerbate soil erosion, degrade surface and groundwater runoff, and reduce biodiversity, leading to significant carbon loss from soil (Brye, 2006). The mining disturbance gradient also negatively affects soil microbial diversity, taxonomic composition, and ecological distribution (Xiao et al., 2021). Despite decades of restoration efforts, full recovery to baseline conditions remains difficult (de Quadros et al., 2016). Therefore, research on the impacts of underground mining on surface ecological environments, particularly on SOC stability and microbial metabolism, is essential to advance sustainable development and achieve carbon neutrality in mining areas.

Biological soil crusts (biocrusts), formed by complex communities of cyanobacteria, microalgae, bacteria, microfungi, lichens, and mosses, play critical roles in arid and semiarid ecosystems (Bowker et al., 2013). These crusts interact with soil particles through mycelia, rhizoids, and exudates, thereby facilitating soil stabilization, nutrient cycling, and organic matter accumulation (Phillips et al., 2022). In coal mining areas, biocrusts develop in response to harsh conditions typical of post-mining environments, which often include nutrient-poor soils exposed to extreme irradiance, UV radiation, drought, temperature fluctuations, and wind and water erosion (Lukešová, 2001). Biocrusts are particularly effective at stabilizing soil surfaces, preventing erosion, and improving soil quality, especially in coarse-textured soils prone to erosion because they lack natural physical crusts. Moreover, biocrusts significantly improve the water-holding capacity and structural stability of the underlying sand (Eldridge et al., 2020). A recent study demonstrated that *S. caninervis*, a desert moss adapted to a wide range of extreme environments, exhibits remarkable resistance to gamma irradiation and can survive while maintaining vitality under simulated Martian conditions (Li et al., 2024). In some cases, biocrusts can alter water infiltration rates by increasing surface roughness, reducing runoff, and enhancing water retention for plant use (Sun et al., 2004; Spröte et al., 2010). In addition to these protective functions, biocrusts contribute significantly to the SOC pool in arid and semi-arid ecosystems. Through photosynthesis, biocrusts fix approximately 0.59 Pg C yr^–1^, making them a major source of carbon in these regions (Elbert et al., 2012). Although several studies have focused on carbon fixation, respiration, and SOC accumulation in biocrusts (Kheirfam, 2020; Xu et al., 2022; Xie et al., 2023), uncertainties remain regarding the effects of biocrusts on different SOC fractions and their stabilities, particularly in coal mining subsidence areas. SOC can be categorized into microbial biomass carbon (MBC), dissolved organic carbon (DOC), easily oxidized organic carbon (EOC), and recalcitrant organic carbon (ROC) based on the average residence time of organic carbon in soil (Ma et al., 2020). DOC is considered the primary energy source for soil microbes and is an indicator of carbon availability (Kalbitz et al., 2000), while MBC, though a small fraction of SOC, significantly influences microbial processes (Joergensen and Wichern, 2018). The stability of SOC pools was directly influenced by changes in these fractions. Therefore, understanding the composition and stability of organic carbon in biocrusts is crucial for accurately assess its role in regulating the carbon cycle in coal-mining subsidence areas.

Extracellular enzyme activity and enzyme stoichiometry in the C, N, and P cycles are widely used to evaluate the balance between microbial nutrient demand and environmental nutrient availability (Moorhead et al., 2013). These enzyme activities provide insights into the nutrient transformations carried out by biocrusts, with potential extracellular enzyme activity serving as a key indicator of microbial processes (Dove et al., 2020). Studies have shown that soil enzyme activities, including β-glucosidase, sucrose, and urease, decrease significantly 1 year after the removal of moss-forming biocrusts relative to intact biocrusts (Cheng et al., 2021). In desert ecosystems, the spatial distribution of biocrusts and soil depth play a crucial role in shaping nutrient dynamics and enzyme activity. Biocrust microorganisms are more constrained by P, whereas soils beneath biocrusts show stronger N limitation. This disparity is primarily due to the substantial carbon and nitrogen inputs from moss biocrusts, which supply vital nutrients to microbial communities (Huang et al., 2024). Additionally, coal mining disturbances have been shown to affect the microbial activity in biocrusts. Increased microbial activity following mining disturbances is correlated with enhanced nutrient availability, which, in turn, promotes microbial metabolism (Dal Bello et al., 2021; Guo L. et al., 2024). However, the specific mechanisms by which varying coal mining disturbance gradients influence microbial metabolic processes and nutrient limitations within biocrusts remain poorly understood.

Carbon use efficiency (CUE) is a key parameter in microbially mediated carbon cycling, describing the proportion of assimilated carbon allocated to microbial biomass rather than lost through respiration. As such, the CUE integrates microbial growth and respiration processes and reflects the dynamic balance between SOC sequestration and release (Tao et al., 2023). A higher CUE indicates more efficient carbon retention within the microbial biomass, whereas a lower CUE implies greater carbon loss via respiratory pathways (Wang et al., 2018). Soil microbial CUE is regulated by several factors. In addition to soil nutrient stoichiometry, ecosystem-specific environmental conditions, including climate and inherent soil properties, strongly influence microbial metabolic strategies and carbon allocation patterns (Manzoni et al., 2012). Using enzyme stoichiometry approaches, He et al. (2023) demonstrated that microbial carbon limitation was the dominant constraint on CUE across farmland, forest, and grassland ecosystems. In biocrust-dominated systems, Guo Y. et al. (2024) reported that alleviating microbial P limitation increased microbial CUE, thereby contributing to increased carbon accumulation in the subsoil beneath moss biocrusts. Despite these advances, the influence of coal mining on CUE within biocrusts remains incompletely characterized. In particular, how abiotic changes associated with different mining disturbance gradients interact with biocrust biological processes to influence the microbial CUE remain poorly understood.

In coal-mining subsidence areas, we linked the ecoenzymatic stoichiometry model to biocrust characteristics and organic carbon fractions to explore the effects of mining disturbance gradients on biocrust carbon stability and on microbial metabolic limitations. We also tested whether relationships existed among vegetation types (herbaceous and shrub), biocrusts properties, and microbial resource limitations across different mining disturbance gradients. We hypothesized that coal mining disturbances would alter the distribution of moss and cyanobacterial biocrusts, which in turn would affect nutrient cycling and photosynthetic pigment content; decrease the organic carbon fractions and stability of biocrusts; and reduce extracellular enzyme activities, thereby exacerbating microbial nutrient limitations in the biocrusts. To test these hypotheses, we focused on moss and cyanobacterial biocrusts in shrub and herbaceous treatments across mining-disturbance gradients. By analyzing and comparing nutrient cycling, organic carbon stability, and extracellular enzyme activities in these biocrusts, we aimed to identify the key factors that influence carbon stability and microbial nutrient limitations. We anticipate that this study will contribute to the development of effective ecological restoration strategies for coal mining subsidence areas and expand our understanding of biocrust ecology in arid and semi-arid environments.

## 2 Materials and methods

### 2.1 Study site description and experimental sampling

**FIGURE 1:**
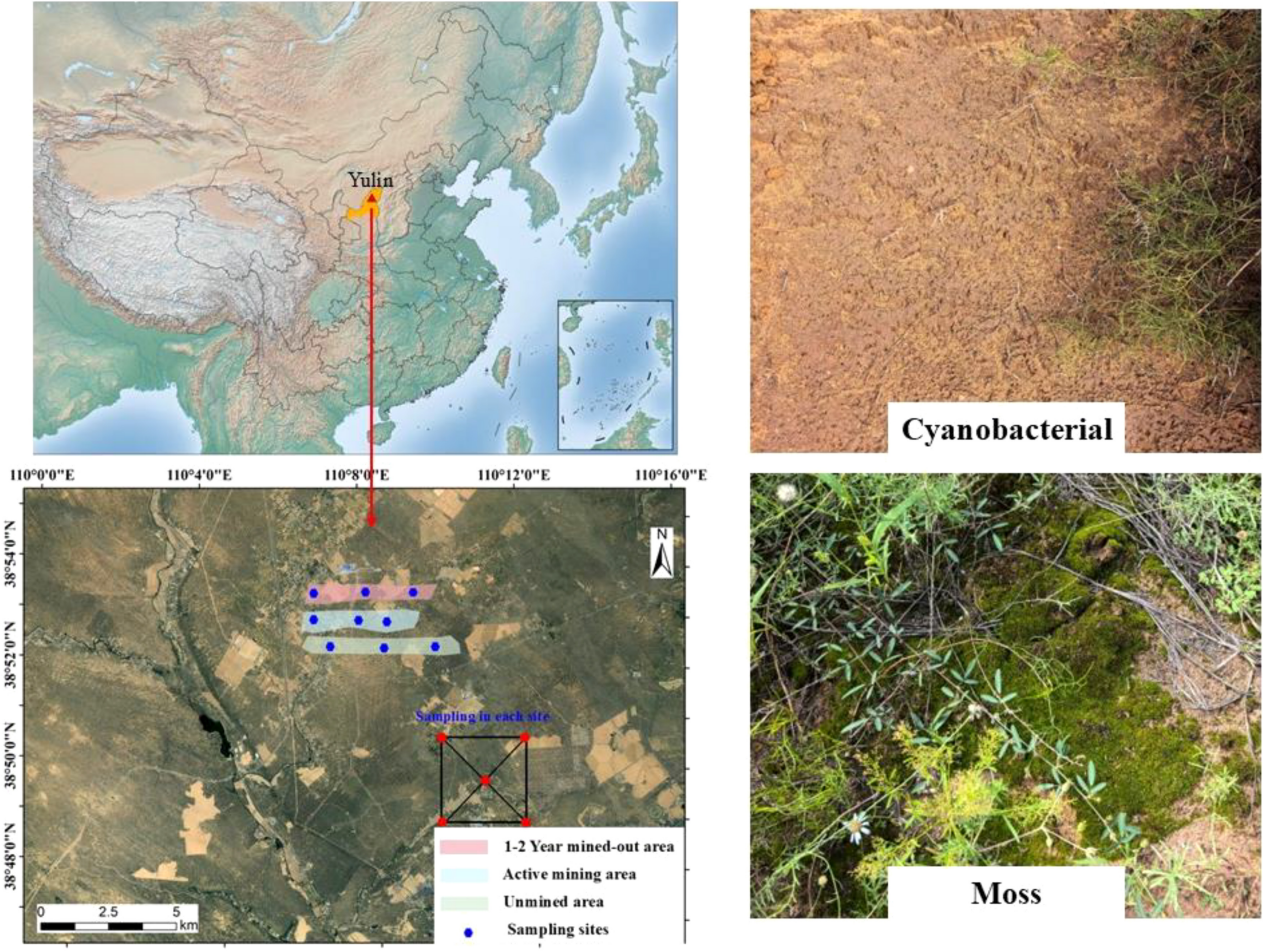
Location of study site.

The study was conducted in the Jinjie coal mining subsidence area (38°50′-38°54′N, 110°2′-110°16′E), located in Yulin County, Shaanxi Province, northwest China (Figure 1). The region experiences a semi-arid continental monsoon climate with an average annual temperature of 8.5°C. The climate is characterized by pronounced temperature variability, low precipitation, high evaporation, and irregular precipitation distribution. Approximately 60% of annual precipitation occurs between the 7th and 9th months. This area typically freezes in October and remains frozen until April, resulting in a short frost-free period. Over the years, the average annual precipitation has been 435.7 mm, whereas the average annual evaporation has been 2111.2 mm. The dominant soil type was loose Arenosols (FAO/UNESCO classification). Extensive underground mining activities have led to land subsidence, collapse, and numerous permanent cracks. Based on the chronological mining sequence, this study designated the 2023 working face areas as 1–2 year mined-out zones, the 2025 working face areas as active mining zones, and the unmined (planned) areas as control zones (Figure 1).

The plant community is composed of trees, shrubs and herbaceous species. The dominant tree species include *Pinus sylvestris var. mongholica Litv.*, and *Populus przewalskii Maxim.*, while the main herbaceous species are *Artemisia desertorum Spren*g., *Artemisia capillaris Thunb*., and *Elymus dahuricus Turcz*. The shrub species included *Caragana korshinskii Kom*., *Salix cheilophila*., and *Tamarix chinensis Lour*. Initial field investigations revealed that moss and cyanobacterial biocrusts are widely distributed in both shrub- and herbaceous-domianted vegetation. Consequently, these two vegetation types were selected as study plots for examining biocrust characteristics. The moss cover ranged from 15.6 to 26.8%, whereas the cyanobacterial cover varied from 9.2 to 18.4%, both of which occupy significant ecological niches.

Sampling was conducted in May 2025 across three gradients of coal mining disturbances (unmined area, active mining area, and 1–2 year mined-out area; Figure 1). Within each disturbance gradient, twenty 25 × 25 cm plots were randomly established to assess vegetation and biocrust cover. The percentage cover of vascular plants and biocrusts was quantified using a point-intercept approach with a 25 × 25 cm gridded quadrat, and the mean cover values are summarized in Table 1. In addition, three 20 × 20 m plots were randomly selected within each disturbance gradient. To minimize topographic effect, adjacent plots were separated by at least 500 m and were comparable in slope, aspect, and elevation. Within each 20 × 20 m plot, three 1 × 1 m subplots were designated for sampling the understory herbaceous vegetation. The collected plant material was blanched, oven-dried, and ground to pass through a 0.25 mm sieve before determining the concentrations of carbon, nitrogen, phosphorus, and potassium. Biocrust samples were collected from each subplot following a five-point composite sampling design, in which five subsamples were combined to form one representative sample per plot. A total of 18 biocrust samples were collected (three disturbance gradients × two vegetation types × three replicates). The biocrusts were carefully excavated to their natural thickness (1–9 mm), using sterile tools. Samples were passed through a 2 mm sieve to remove plant roots, stones, and coarse debris. Each biocrust sample was subdivided into three portions: one air-dried for physicochemical analyses, a second stored at −20°C for biological measurements, and a third preserved at −80°C for extracellular enzyme activity assays. Concurrently, intact moss-dominated and cyanobacteria-dominated biocrusts were collected using sterile 9-cm-diameter Petri dishes across all disturbance gradients and vegetation types. Three replicate samples were obtained per treatment for each biocrust type. These samples were shade-dried and subsequently used to determine biocrust thickness, biomass, bulk density, and photosynthetic pigment content.

**TABLE 1:** Effects of mining disturbance gradients on surface composition characteristics.

Surface composition characteristics	Unmined area		Active mining area		1–2 year mined-out area		G (2)		V (1)		G × V (2)	
	Herbaceous	Shrub	Herbaceous	Shrub	Herbaceous	Shrub	*F*	*P*	*F*	*P*	*F*	*P*
Vegetation cover (%)	26.4 ± 3.9^a^	23.2 ± 3.2^b^	20.8 ± 1.7^bc^	19.6 ± 3.5^c^	22.8 ± 4.6^bc^	22.4 ± 2.0^bc^	9.7	***	3.5	NS	0.9	NS
Litter cover (%)	25.6 ± 5.1^a^	22.8 ± 2.7^ab^	21.2 ± 1.9^b^	20.4 ± 2.3^b^	22.4 ± 3.9^b^	21.6 ± 2.1^b^	5.9	**	3.2	NS	0.7	NS
Moss cover (%)	26.8 ± 1.9^a^	22.0 ± 3.4^b^	19.6 ± 3.0^b^	15.6 ± 1.3^c^	21.6 ± 2.1^b^	20.0 ± 3.3^b^	34.3	***	26.7	***	2.1	NS
Cyanobacterial biocrust cover (%)	14.0 ± 2.8^b^	18.4 ± 3.8^a^	9.2 ± 1.9^d^	10.8 ± 2.0^cd^	12.4 ± 1.3^bc^	13.6 ± 2.1^b^	45.1	***	20.3	***	3.6	*
Bare soil cover (%)	6.4 ± 2.1^d^	13.2 ± 1.9^c^	22.8 ± 1.9^a^	25.2 ± 5.0^a^	18.8 ± 2.7^b^	20.0 ± 2.6^b^	123.0	***	21.1	***	5.1	*
Understory vegetation carbon (g⋅kg^–1^)	376.9 ± 0.5^a^	370.9 ± 0.7^b^	356.4 ± 0.3^e^	345.3 ± 0.2^f^	365.2 ± 0.6^c^	360.2 ± 0.5^d^	3149.9	***	967.5	***	63.7	***
Understory vegetation nitrogen (g⋅kg^–1^)	9.3 ± 0.07^a^	8.0 ± 0.02^b^	7.1 ± 0.03^e^	6.9 ± 0.02^f^	7.6 ± 0.04^c^	7.34 ± 0.01^d^	3073.9	***	1073.9	***	375.6	***
Understory vegetation phosphorus (g⋅kg^–1^)	2.1 ± 0.02^a^	2.0 ± 0.01^b^	1.3 ± 0.01^e^	1.2 ± 0.01^f^	1.7 ± 0.01^c^	1.6 ± 0.02^d^	3821.6	***	298.4	***	11.0	***
Understory vegetation potassium (g⋅kg^–1^)	36.3 ± 0.1^a^	34.6 ± 0.2^b^	28.4 ± 0.2^e^	26.8 ± 0.1^f^	33.5 ± 0.1^c^	31.6 ± 0.1^d^	6432.9	***	898.9	***	2.3	NS
Moss thickness (mm)	9.3 ± 0.2^a^	8.5 ± 0.8^ab^	8.2 ± 1.1^b^	7.3 ± 0.4^c^	8.9 ± 0.9^ab^	7.9 ± 0.1^bc^	7.2	***	12.6	***	0.1	NS
Cyanobacterial thickness (mm)	5.6 ± 0.5^a^	5.8 ± 0.4^a^	4.4 ± 0.2^d^	4.7 ± 0.6^cd^	5.0 ± 0.4^bc^	5.4 ± 0.2^ab^	20.8	***	4.1	NS	0.2	NS

Significant differences (*P* < 0.05) within a row are represented by different lowercase letters, based on Duncan’s multiple range test. Data are mean ± standard deviation. The abbreviations “G,” “V,” and “G × V” indicate individual and interaction effects of different gradients of mining disturbance and the two vegetation types. ***P < 0.001; **P < 0.01; *P < 0.05; NS no significant difference. The same convention applies hereafter.

### 2.2 Understory vegetation nutrient analysis, biocrust characteristics, and photosynthetic pigments

The carbon content of understory vegetation was determined using the potassium dichromate external heating method. Total nitrogen was measured by the Kjeldahl method following digestion with an H_2_SO_4_–H_2_O_2_ mixture. Phosphorus and potassium concentrations were quantified by inductively coupled plasma optical emission spectrometry (ICP-OES) after digestion with HNO_3_–H_2_O_2_. The biocrust thickness and bulk density were determined using a Vernier caliper and the soil coating method, respectively. The biomass of the moss-dominated biocrusts was quantified gravimetrically. Briefly, the biocrust samples were first moistened by spraying with distilled water to facilitate separation. A subsample with a fixed area of 0.95 cm^2^ was then excised and gently washed with distilled water through a 0.25 mm mesh sieve to remove mineral particles. The retained moss material was collected and oven-dried at 65°C to constant mass. Moss biocrust biomass (g⋅dm^–2^) was calculated by normalizing the dry mass (g) to the sampled surface area (0.95 cm^2^ converted to dm^2^).

The biomass of cyanobacteria-dominated biocrusts was quantified using an ethanol extraction method and expressed as chlorophyll (Chl) *a* content (Castle et al., 2011). Cyanobacterial biocrust samples were ground and incubated in the dark to prevent pigment degradation. An accurately weighed subsample (3.000 ± 0.001 g, M) was extracted with 6 mL of ethanol (V). The absorbance of the extract was measured at 665 nm and 750 nm using a spectrophotometer, and the corresponding values were recorded as E_665_ and E_750_. Subsequently, the extract was acidified with HCl, and after 2 min, absorbance was measured again at the same wavelengths, yielding A_665_ and A_750_. Chl *a* concentration (mg⋅g ^–1^) was calculated according to (Equation 1):


$$\text{Chl}\,a=29.6\times \left[\left(E_{665}-E_{750}\right)-\left(A_{665}-A_{750}\right)\right]\times V/M$$ (1)

where V is the extraction volume (mL), and M is the fresh mass of the biocrust sample (g).

The Chl *a*, Chl *b*, and carotenoids contents in the moss-dominated biocrusts were determined by ethanol extraction followed by spectrophotometric analysis (Wellburn and Lichtenthaler, 1984). Briefly, 0.05 g of fresh moss tissue was finely ground in a mortar, and photosynthetic pigments were extracted using 95% (v/v) ethanol. The extract was heated in a water bath at 85°C for 5 min to ensure complete pigment extraction, then centrifugation at 4,000 rpm for 10 min at room temperature. The absorbance of the supernatant was measured at 665, 649, and 470 nm using a UV–Vis spectrophotometer, and the corresponding absorbance values were recorded as A_665_, A_649_, and A_470_, respectively. Pigment concentrations (mg⋅L^–1^) were calculated according to (Equations 2–4):


Chla=13.95A−6656.88A649 (2)

Chlb=24.96A−6497.32A665 (3)

Carotenoids=(1000A−4702.05Chlα−114.8Chlb)/245 (4)

Carotenoids and scytonemin in cyanobacteria-dominated biocrusts were extracted using acetone and quantified by spectrophotometry following established protocols (Garcia-Pichel and Castenholz, 1991). Briefly, a 1 × 1 cm section of air-dried cyanobacterial biocrust was finely ground, extracted with acetone, and incubated in darkness at 4°C overnight. The extract was subsequently filtered through a Whatman No. 5 filter paper. Absorbance of the filtrate was measured at 384, 490, and 663 nm using a UV–Vis spectrophotometer, and the corresponding absorbance values were recorded as A384, A490, and A663, respectively. Scytonemin and carotenoid contents were expressed on an area basis (A⋅cm^–2^) and calculated according to (Equations 5, 6) as follows:


Scytonemin=1.04A−3840.79A−6630.27A490 (5)

Carotenoids=1.02A−4900.08A−3840.026A663 (6)

### 2.3 Physicochemical and biological properties of biocrusts

The pH and electrical conductivity (EC) of the biocrusts were measured in soil–water suspensions at ratios of 1:2.5 and 1:5 (w/v), respectively, using a pH/EC meter (SevenDirect, Mettler Toledo, Switzerland). Total nitrogen (TN) in biocrusts was determined using the Kjeldahl digestion and distillation method, while total phosphorus (TP) was quantified by ICP-OES following digestion with an H_2_SO_4_–HClO_4_ mixture. Ammonium nitrogen (NH4 ^+^–N) and nitrate nitrogen (NO_3_^+^–N) were extracted with 1 mol⋅L^–1^ KCl solution and measured using a continuous flow analyzer (AA3, Bran + Luebbe, Norderstedt, Germany). Microbial biomass carbon (MBC), nitrogen (MBN), and phosphorus (MBP) were determined using the chloroform fumigation–extraction method. For MBC and MBN analyses, the biocrust samples were divided into two subsamples: a non-fumigated control and a chloroform-fumigated treatment, both incubated in the dark for 7 days. Following incubation, 8.0 g of non-fumigated and fumigated samples were extracted with 0.5 mol⋅L^–1^ K_2_SO_4_. The extracts were filtered and analyzed for organic carbon and total nitrogen using a TOC/TN elemental analyzer (Vario Max CN, Elementar, Hanau, Germany). MBP was determined using a similar fumigation–extraction procedure. Paired non-fumigated and fumigated subsamples (3.0 g each) were extracted, filtered, and analyzed for phosphorus concentration using ICP-OES. Dissolved organic nitrogen (DON) was determined by extracting non-fumigated samples with 0.5 mol⋅L^–1^ K_2_SO_4_ and analyzing the extracts using the TOC/TN analyzer. Available phosphorus (AP) was measured following extraction of non-fumigated samples with 0.5 mol⋅L^–1^ NaHCO_3_ and subsequent determination by ICP-OES. Microbial biomass C, N, and P were calculated as the differences between the nutrient concentrations extracted from the fumigated and non-fumigated samples.

### 2.4 Organic carbon fractions and extracellular enzyme activities of biocrusts

The organic carbon content of the biocrusts was quantified by potassium dichromate oxidation method. ROC was determined using the hydrochloric acid digestion method (Yang et al., 2019). Briefly, 2.0 g of air-dried soil, sieved through a 1 mm mesh, was placed in a 100 mL hard glass test tube. To this, 5 mL of 6 mol⋅L^–1^ HCl solution was added, and the mixture was sterilized at 125°C for 16 h. After sterilization, the sample was transferred to a 50 mL centrifuge tube, and 30 mL of distilled water was added. The sample was shaken thoroughly and centrifuged at 4,000 rpm for 3 min. The supernatant was discarded, and the washing step was repeated five times with fresh distilled water until all HCl was removed. The washed soil was dried at 55°C and subsequently treated with concentrated H_2_SO_2_ and K_2_Cr_2_O_7_ to determine ROC using the external heat capacity method, consistent with the procedure for measuring SOC. DOC was extracted from unfumigated soil samples using 0.5 mol⋅L^–1^ potassium sulfate and analyzed using a TOC/TN elemental analyzer (Vario Max-CN, Elementar, Hanau, Germany). EOC was measured by the 333 mM potassium permanganate oxidation method, as described by Sun et al. (2023).

The ratio of EOC to SOC is an indicator of the activity of SOC. A higher EOC/SOC ratio indicates increased microbial activity, thereby facilitating the decomposition and utilization of organic carbon in the soil. In contrast, the ROC-to-SOC ratio reflects the stability of SOC. A higher ROC/SOC ratio indicates greater stability, making the organic carbon more resistant to microbial decomposition and utilization. Additionally, the EOC-to-ROC ratio provided further insight into the stability of the SOC. A higher proportion of EOC signifies lower stability, while a greater content of ROC suggests increased stability and resistance to decomposition.

Ecological studies typically quantify only the enzyme activities of terminal reactions that produce assimilable products from major carbon, nitrogen and phosphorus sources. The most widely measured extracellular enzyme activities include carbon acquisition enzymes [β-1,4-glucosidase (BG), β-1,4-xylosidase (BX) and β-D-cellulodisaccharide hydrolase (CBH)]; and nitrogen acquisition enzymes [β-1,4-N-acetylglucosaminidase (NAG) and L-leucine aminopeptidase (LAP); and phosphorus acquisition enzyme (alkaline phosphatase (AP)]. Extracellular enzyme activity was determined as described by Saiya-Cork et al. (2002). Briefly, 1 g of fresh soil sample was mixed with 125 mL of Tris-HCl buffer (pH = 8) and shaken for 2 h at 25°C and 180 rpm to prepare a soil suspension. A 1 mL aliquot of the soil suspension was transferred to 96-well microplates. The corresponding fluorescent substrate for each enzyme was immediately added to the sample wells, and each sample was analyzed in duplicate. Blank wells consisting of soil suspension plus Tris-HCl buffer, as well as quenched standard sample wells, were included. 4-amino-4-methylcoumarin (MUB) was used as the fluorescent substrate for BG, BX, CBH, NAG, and AP, and 7-amino-4-methylcoumarin (MUC) was used as the LAP. The microplates were then incubated in the dark for 25 h. After incubation, fluorescence intensity was measured using a microplate reader (Tecan Infinite M200, Salzburg, Austria) with excitation at 365 nm and emission at 450 nm. Enzyme activity was expressed as micromoles of substrate released per gram of soil organic matter per hour (μmol⋅g SOM^–1^⋅h ^–1^).

### 2.5 Microbial metabolic limitations

Enzymatic stoichiometry analysis was employed to calculate the length of the microbial metabolic restriction vector (unitless), which represented the relative carbon limitation (C limit) of the microorganisms, and the vector angle (°), which indicated N or P limitation. Angles >45° represent phosphorus limitation, while angles <45° indicate nitrogen limitation (Moorhead et al., 2016). The formulas for calculating vector length and angle are as shown in Equations 7–11:


Vector⁢length= (7)

$$\sqrt{{\left[\frac{\mathrm{BG}+\mathrm{CBH}}{\mathrm{BG}+\mathrm{CBH}+\mathrm{AP}}\right]}^{2}+{\left[\frac{\mathrm{BG}+\mathrm{CBH}}{\mathrm{BG}+\mathrm{CBH}+\mathrm{NAG}+\mathrm{LAP}}\right]}^{2}}$$

Vector⁢angle=DEGREES (8)

$$\left[\mathrm{ATAN2}\,\left(\frac{\mathrm{BG}+\mathrm{CBH}}{\mathrm{BG}+\mathrm{CBH}+\mathrm{AP}}\right),\left(\frac{\mathrm{BG}+\mathrm{CBH}}{\mathrm{BG}+\mathrm{CBH}+\mathrm{NAG}+\mathrm{LAP}}\right)\right]$$

The biogeochemical equilibrium model was applied to determine CUE (Sinsabaugh et al., 2016):


CUE=CUE×max[(B/C:NL×C:N1/EEA)C:N× (9)

(B/C:PL×C:P1/EEA)C:P]/

[(K+C:NS)C:N×(K+C:PS)C:P]0.5

Where S_C:N_ and S_C:P_ represent the microbial biomass ratios of nitrogen and phosphorus, respectively, calculated as follows:


S=C:NB/C:NL×C:N1/EEAC:N (10)

S=C:PB/C:PL×C:P1/EEAC:P (11)

where EEA_C:N_ and EEA_C:P_ are the nitrogen- and phosphorus-acquisition enzyme ratios, respectively. L_C:N_ and L_C:P_ represent the C:N and C:P ratios of the available nutrients, respectively. B_C:N_ and B_C:P_ represent the microbial biomass ratios of carbon, nitrogen, and phosphorus. K_C:N_ and K_C:P_ are the semi-saturation constants for the availability of nitrogen and phosphorus (both set to 0.5). Finally, CUEmax represents the upper limit of microbial growth efficiency, assumed to be 0.6.

### 2.6 Statistical analysis

Differences in biocrust characteristics, photosynthetic pigments, physicochemical properties, organic carbon fractions, and extracellular enzyme activities across different mining disturbance gradients and vegetation types were analyzed using one-way analysis of variance (ANOVA) in IBM SPSS Statistics v. 24. *Post-hoc* comparisons of the mean values were performed using Duncan’s multiple range test. A two-way ANOVA was employed to examine the interactive effects of mining disturbance gradients and vegetation types on surface composition, biocrust properties, photosynthetic pigments, physicochemical characteristics, organic carbon fractions, extracellular enzyme activities, and CUE. Spearman’s correlation analysis and Mantel tests were conducted to explore the relationships among understory vegetation, biocrust properties, photosynthetic pigments, soil nutrients, microbial biomass, soil organic carbon stability, microbial nutrient limitation, and CUE. These analyses were conducted using R software with the “ggcor” package for visualization. Additionally, the relative importance of predictor variables was assessed using the “relaimpo” package in R. Partial least squares path modeling (PLS-PM) was performed to further investigate the direct and indirect pathways through which various factors influence microbial CUE in biocrusts. The models were constructed using the “innerplot” function of the “plspm” R package.

## 3 Results

### 3.1 Surface composition and physicochemical properties of biocrusts

Mining disturbance gradients, vegetation types, and their interactions significantly affected changes in surface understory vegetation and biocrusts composition (*P* < 0.05, Table 1). Vegetation and litter cover were significantly higher in the herbaceous treatment of unmined areas than in the herbaceous and shrub treatments of active mining and 1–2 year mined-out areas. The biocrusts cover ranged from 26.4 to 40.8%. Within this range, moss cover was significantly higher in the herbaceous treatment of unmined areas than in all other treatments (i.e., shrub treatment of unmined areas and both herbaceous and shrub treatments of active mining and 1–2 year mined-out areas). In contrast, cyanobacterial cover was significantly greater in the shrub treatment of unmined areas than in all other treatments. Understory vegetation carbon, nitrogen, phosphorus, and potassium showed a consistent pattern—concentrations in the herbaceous treatment of the unmined areas were significantly higher than in all other treatments. The greatest thickness of biocrusts was observed in unmined areas, followed by 1–2-year mined-out and active-mining areas.

Mining disturbance gradients had a greater effect on the physicochemical properties and nutrient content of biocrusts across vegetation types (Supplementary Table 1). The pH and EC of the cyanobacterial biocrust were significantly higher than those of the moss biocrust. The highest pH and EC were found in the cyanobacterial biocrust herbaceous treatment in active mining areas, and the lowest were observed in the moss biocrust herbaceous treatment in unmined areas. The moss biocrust TN, TP, AP, NO_3_^–^-N, NH_4_^+^-N, DON, MBN, and MBP were significantly higher than those of the cyanobacterial biocrust soil across vegetation treatments with different mining-disturbance gradients. The highest TN, TP, AP, NO_3_^–^-N, NH_4_^+^-N, DON, MBN, and MBP were observed in the moss biocrust herbaceous treatment of unmined areas, which were 18.8–36.2, 26.7–54.1, 9.8–23.5, 17.0–45.5, 9.5–23.8, 3.5–10.9, 14.5–20.8, and 14.3–18.3% higher than those in the moss biocrust herbaceous treatment of active mining and 1–2 year mined-out areas, respectively.

### 3.2 Biocrusts characteristics and photosynthetic pigments

Except for biomass, biocrusts characteristics and photosynthetic pigments were significantly influenced by the main and interactive effects of mining disturbance gradient and vegetation type (*P* < 0.05; Table 2). Moss biomass was substantially higher in unmined areas than in active and 1–2 year mined-out areas, with the highest values observed in the herbaceous treatment of unmined areas. Cyanobacterial biomass was significantly higher in the shrub treatments than in the herbaceous treatments, with the highest and lowest values observed in the shrub treatments of unmined areas and herbaceous treatments of active mining areas, respectively. Furthermore, the content of moss Chl *a* and *b*, carotenoids, and bulk density were significantly higher in the herbaceous treatments than in the shrub treatments. Peak values were observed in the herbaceous treatment of unmined areas, exceeding those in the shrub treatment of active mining areas by 35.8, 176.1, 69.6, and 45.4%. Cyanobacterial carotenoids, scytonemin, and bulk density exhibited consistent gradients across the mining disturbance gradients. Their values were significantly higher in unmined areas, intermediate in the 1–2 year mined-out areas, and lowest in active mining areas. Furthermore, these parameters were consistently higher in the shrub treatments than in the herbaceous treatments in all areas.

**TABLE 2:** Effects of mining disturbance gradients on biocrust characteristics and photosynthetic pigments.

Biocrust properties	Unmined area		Active mining area		1–2 Year mined-out area		G (2)		V (1)		G × V (2)	
	Herbaceous	Shrub	Herbaceous	Shrub	Herbaceous	Shrub	*F*	*P*	*F*	*P*	*F*	*P*
Moss biomass (g⋅dm^–2^)	0.09 ± 0.01^a^	0.08 ± 0.01^ab^	0.07 ± 0.01^b^	0.05 ± 0.01^c^	0.08 ± 0.01^ab^	0.07 ± 0.01^b^	21.9	***	7.4	**	0.7	NS
Cyanobacterial biomass (mg⋅g^–1^)	15.5 ± 0.3^b^	17.0 ± 0.6^a^	11.6 ± 0.4^e^	12.7 ± 0.8^d^	14.5 ± 0.4^c^	15.6 ± 0.8^b^	164.7	***	41.6	***	0.6	NS
Moss chlorophyll *a* (mg⋅g^–1^)	4.25 ± 0.02^a^	3.97 ± 0.02^b^	3.56 ± 0.01^d^	3.13 ± 0.02^e^	3.84 ± 0.03^c^	3.64 ± 0.14^d^	250.1	***	120.4	***	5.5	**
Moss chlorophyll *b* (mg⋅g^–1^)	3.12 ± 0.04^a^	2.74 ± 0.02^b^	1.35 ± 0.02^e^	1.13 ± 0.05^f^	2.57 ± 0.05^c^	2.23 ± 0.05^d^	2785.6	***	271.7	***	7.0	**
Moss carotenoids (mg⋅L^–1^)	1.34 ± 0.03^a^	1.25 ± 0.01^b^	0.93 ± 0.01^e^	0.79 ± 0.01^f^	1.12 ± 0.01^c^	0.99 ± 0.01^d^	1456.4	***	331.8	***	6.8	**
Cyanobacterial carotenoids (A⋅cm^–2^)	0.074 ± 0.01^b^	0.09 ± 0.01^a^	0.049 ± 0.01^e^	0.052 ± 0.01^d^	0.056 ± 0.01^d^	0.065 ± 0.01^c^	464.8	***	148.5	***	5.1	**
Cyanobacterial Scytonemin (A⋅cm^–2^)	0.093 ± 0.01^b^	0.111 ± 0.02^a^	0.050 ± 0.01^e^	0.066 ± 0.01^d^	0.084 ± 0.01^c^	0.085 ± 0.01^c^	351.1	***	71.2	***	17.0	***
Moss bulk density (g⋅cm^–3^)	4.26 ± 0.02^a^	4.04 ± 0.03^b^	3.23 ± 0.01^e^	2.93 ± 0.01^f^	3.71 ± 0.01^c^	3.50 ± 0.01^d^	7325.3	***	1181.7	***	16.9	***
Cyanobacterial bulk density (g⋅cm^–3^)	2.05 ± 0.04^b^	2.23 ± 0.01^a^	1.67 ± 0.01^f^	1.71 ± 0.01^e^	1.80 ± 0.01^d^	1.89 ± 0.01^c^	1140.3	***	182.9	***	23.9	***

Significant differences (*P* < 0.05) within a row are represented by different lowercase letters, based on Duncan’s multiple range test. Data are mean ± standard deviation. The abbreviations “G,” “V,” and “G × V” indicate individual and interaction effects of different gradients of mining disturbance and the two vegetation types. ***P < 0.001; **P < 0.01; NS no significant difference. The same convention applies hereafter.

### 3.3 Biocrusts organic carbon fractions and stability

**FIGURE 2:**
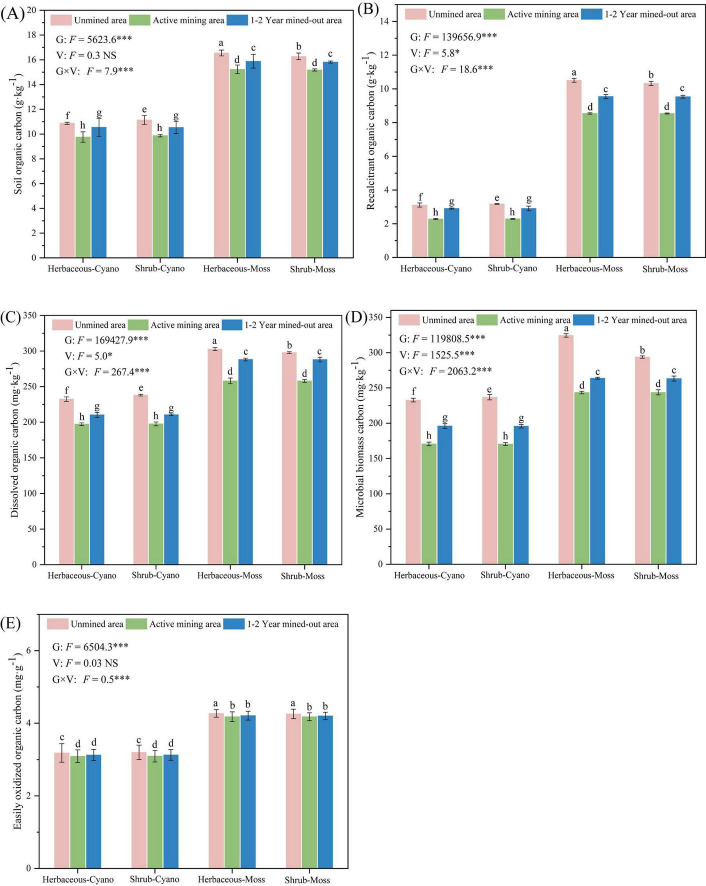
Effects of mining disturbance gradients on biocrust organic carbon fractions. **(A)** Soil organic carbon, **(B)** recalcitrant organic carbon, **(C)** dissolved organic carbon, **(D)** microbial biomass carbon, **(E)** easily oxidized organic carbon. Data are mean ± standard deviation. Bars with different letters indicate significant differences at *P* < 0.05. The abbreviations “G,” “V,” and “G × V” indicate individual and interaction effects of different gradients of mining disturbance and the two vegetation types. ***P < 0.001; *P < 0.05; NS no significant difference. Cyano, Cyanobacterial.

Active mining significantly reduced the organic carbon, ROC, DOC, MBC, and EOC contents in the herbaceous and shrub treatments (Figure 2). Specifically, compared to the unmined areas that received the same treatment, active mining reduced the organic carbon contents of moss in herbaceous treatments, cyanobacterial in herbaceous treatments, moss in shrub treatments, and cyanobacteria in shrub treatments by 8.67, 11.37, 7.25, and 12.87%, respectively (Figure 2A). The ROC, DOC, and MBC contents of the biocrusts in the herbaceous and shrub treatments showed a consistent gradient across the mining disturbance gradients (Figures 2B–D). Biocrusts ROC, DOC, and MBC contents were significantly lower in active mining/1–2 year mined-out areas than in unmined areas, and herbaceous treatment significantly increased moss biocrusts ROC, DOC, and MBC contents, whereas shrub treatment significantly increased cyanobacterial biocrusts ROC, DOC, and MBC contents. Biocrusts EOC content in unmined area was significantly higher than that in active mining/1–2 year mined-out areas for both the herbaceous and shrub treatments. However, there was no significant difference between the active mining area and the 1–2 year mined-out area (Figure 2E). Overall, mining disturbance gradients, as well as their interaction with vegetation type, significantly influenced organic carbon, ROC, DOC, MBC, and EOC contents (*P* < 0.001).

Consistent with our hypothesis, coal mining significantly reduced the stability of organic carbon in biocrusts (Table 3). Cyanobacterial biocrusts EOC/SOC and EOC/ROC were significantly higher by 9.54–15.27 and 143.76–177.14%, respectively, compared to those of moss biocrusts, while moss biocrusts ROC/SOC were significantly higher by 118.48–142.67% than those of cyanobacterial biocrusts. For cyanobacterial biocrusts, the lowest and highest EOC/SOC and EOC/ROC values were observed in the shrub treatment of unmined areas and the herbaceous treatment of active mining areas, respectively. The ROC/SOC ratio in the unmined area was significantly higher than that in the 1–2 year mined-out area, and the lowest in the active mining area. Moreover, the ROC/SOC did not differ significantly between the herbaceous and shrub treatments. For moss biocrusts, the EOC/SOC in the shrub treatment of the unmined area was significantly higher than that in the herbaceous treatment of the unmined areas, but did not differ significantly between the herbaceous and shrub treatments in the active mining/1–2 year mined-out area. Furthermore, the EOC/ROC was highest in the active-mining area but did not differ significantly between the herbaceous and shrub treatments. The ROC/SOC in the unmined area was significantly higher than in the 1–2 year mined-out area, and the lowest was found in the active mining area. Moreover, there were no significant differences in the herbaceous and shrub treatments among these areas.

**TABLE 3:** Effects of mining disturbance gradients on biocrust organic carbon stability.

Disturbance gradients	Vegetation type	Biocrust type	EOC/SOC	ROC/SOC	EOC/ROC
Unmined area	Herbaceous	Cyano	0.293 ± 0.001^d^	0.286 ± 0.002^d^	1.024 ± 0.005^c^
		Moss	0.257 ± 0.004^i^	0.635 ± 0.001^a^	0.406 ± 0.001^g^
	Shrub	Cyano	0.287 ± 0.001^e^	0.285 ± 0.001^d^	1.007 ± 0.008^d^
		Moss	0.262 ± 0.004^h^	0.634 ± 0.001^a^	0.412 ± 0.001^g^
Active mining area	Herbaceous	Cyano	0.317 ± 0.001^a^	0.233 ± 0.003^f^	1.358 ± 0.017^a^
		Moss	0.275 ± 0.007^f^	0.561 ± 0.003^c^	0.490 ± 0.001^e^
	Shrub	Cyano	0.313 ± 0.003^b^	0.232 ± 0.004^f^	1.353 ± 0.023^a^
		Moss	0.275 ± 0.002^f^	0.563 ± 0.004^c^	0.489 ± 0.001^e^
1–2 Year mined-out area	Herbaceous	Cyano	0.296 ± 0.002^c^	0.276 ± 0.003^e^	1.075 ± 0.006^b^
		Moss	0.265 ± 0.003^g^	0.601 ± 0.005^b^	0.441 ± 0.001^f^
	Shrub	Cyano	0.297 ± 0.002^c^	0.276 ± 0.003^e^	1.075 ± 0.001^b^
		Moss	0.266 ± 0.002^g^	0.603 ± 0.003^b^	0.441 ± 0.001^f^
Factor (Df)			*F P*	*F P*	*F P*
G (2)			814.3 ***	21751.8 **	11655.6 ***
V (1)			1.0 NS	0.03 NS	0.9 NS
G × V (2)			5.2 **	0.3 NS	1.1 NS

Significant differences (*P* < 0.05) within a column are represented by different lowercase letters, based on Duncan’s multiple range test. The abbreviations “G”, “V,” and “G × V” indicate individual and interaction effects of different gradients of mining disturbance and the two vegetation types. ***P < 0.001; **P < 0.01; NS, no significant difference.

### 3.4 Biocrusts extracellular enzyme activity and stoichiometry

Across vegetation types, mining disturbance gradients significantly affected extracellular enzyme activities in cyanobacterial and moss biocrusts (*P* < 0.001, Supplementary Figure 1). The extracellular enzyme activities of the moss biocrusts were significantly higher than those of the cyanobacterial biocrusts. Cyanobacterial biocrusts in the shrub treatment of the unmined area exhibited significantly greater C-, N-, and P-acquiring enzyme activities than those in the herbaceous treatment, whereas no such significant differences were found between the shrub and herbaceous treatments in the 1–2 year mined-out and active mining areas. For moss biocrusts, the C-acquiring enzyme activity demonstrated a significant decreasing trend across the increasing mining disturbance gradient, with the unmined area exhibiting the highest activity, followed by the 1–2 year mined-out area, and the active mining area showing the lowest activity (Supplementary Figure 1A). N-acquiring enzyme activity and P-acquiring enzyme activity showed a similar pattern, both of which were highest in the herbaceous treatments of the unmined area, followed by the shrub treatments of the unmined area, and were lowest in the herbaceous and shrub treatments of the active mining area (Supplementary Figures 1B,C).

**FIGURE 3:**
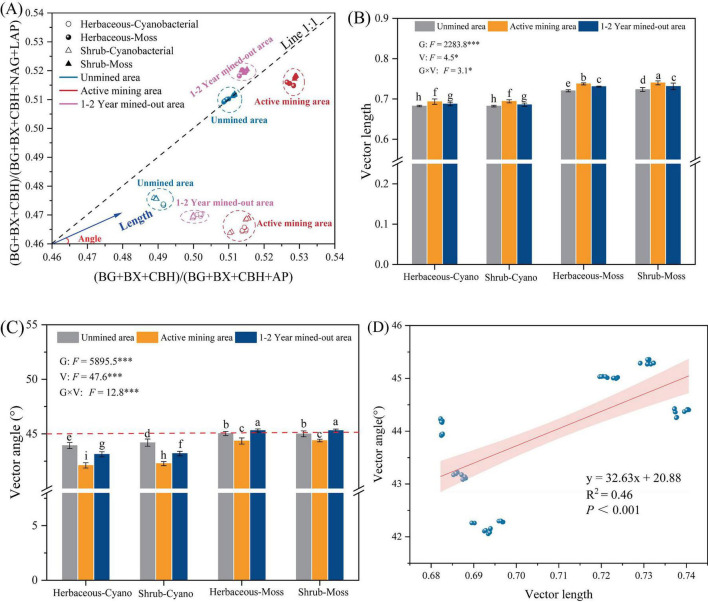
Extracellular enzyme stoichiometry analysis characterizing the ratio of C:N to C:P acquisition **(A)**, presenting the variation in vector length **(B)** and angle **(C)**, and illustrating the relationship between these vector parameters **(D)**. Bars with different letters indicate significant differences at *P* < 0.05. The abbreviations “G”, “V,” and “G × V” indicate individual and interaction effects of different gradients of mining disturbance and the two vegetation types. ***P < 0.001; *P < 0.05.

Mining disturbance gradients and vegetation types exerted significant effects on microbial nutrient metabolism in both cyanobacterial and moss biocrusts (Figure 3). Relative to the 1:1 line, the distribution of points indicated distinct nutrient limitations. Those associated with moss biocrusts in the unmined and 1–2 year mined-out areas (under both herbaceous and shrub treatments) suggested P limitation, whereas points for moss biocrusts in the active mining area and all cyanobacterial biocrusts suggested N limitation (Figure 3A). Furthermore, both mining disturbance gradients and vegetation types, as well as their interaction, significantly influenced the vector length and angle (*P* < 0.05; Figures 3B,C). Vector length (indicative of microbial C limitation) was significantly greater in moss biocrusts than in cyanobacterial biocrusts (Figure 3B). In moss biocrusts, it was highest in active mining areas, intermediate in 1–2 year mined-out areas, and lowest in unmined areas. Furthermore, the shrub treatment exhibited a significantly longer vector length than the herbaceous treatment in both the unmined and active mining areas. A similar trend in vector length across the mining disturbance gradients was observed for both cyanobacterial and moss biocrusts. Consistently, vector length did not differ significantly between shrub and herbaceous treatments in any of the examined areas (unmined, 1–2 year mined-out, and active mining). The vector angle, which ranged from 42.05 to 45.37°, was significantly influenced by the mining disturbance gradient and vegetation type (*P* < 0.001; Figure 3C). Moss biocrusts in the unmined and 1–2 year mined-out areas (under both herbaceous and shrub treatments) exhibited vector angles > 45°, indicating P limitation, with values significantly higher in the 1–2 year mined-out areas than in the unmined areas. In contrast, moss biocrusts in the active mining area and all cyanobacterial biocrusts had vector angles of < 45°, indicating N limitation. For cyanobacterial biocrusts, the vector angle was significantly higher in unmined areas than in 1–2-year mined-out areas, and lowest in active mining areas. Furthermore, the shrub treatment produced a significantly higher vector angle than the herbaceous treatment across all mining-disturbance gradients. Additionally, linear regression demonstrated a significant positive correlation between vector length and angle (*P* < 0.001; Figure 3D).

### 3.5 The interplay between biocrust properties, microbial carbon use efficiency, metabolic limitations, and organic carbon stability

**FIGURE 4:**
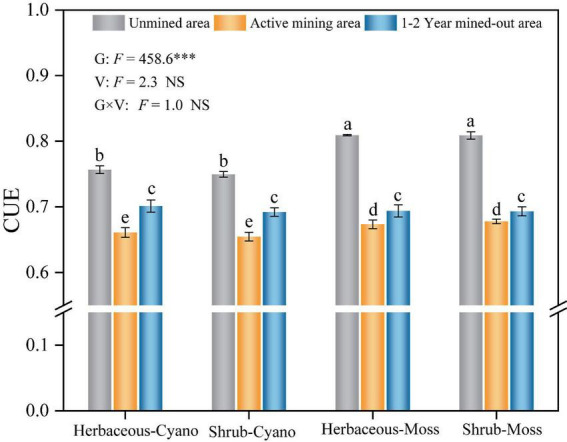
Effects of mining disturbance gradients on biocrust microbial carbon use efficiency. Bars with different letters indicate significant differences at *P* < 0.05. The abbreviations “G”, “V,” and “G × V” indicate individual and interaction effects of different gradients of mining disturbance and the two vegetation types. ***P < 0.001; NS, no significant difference.

The microbial CUE in the biocrusts exhibited significant differences among the mining disturbance gradients (*P* < 0.001; Figure 4). The microbial CUE of biocrusts was significantly higher in unmined areas than in 1–2 year mined-out areas and was lowest in active mining areas. Furthermore, in both unmined and actively mined areas, microbial CUE was significantly higher in moss biocrusts than in cyanobacterial biocrusts, regardless of herbaceous or shrub treatments. In contrast, no significant differences in CUE were observed between moss and cyanobacterial biocrusts in the 1–2 year mined-out areas under either treatment.

**FIGURE 5:**
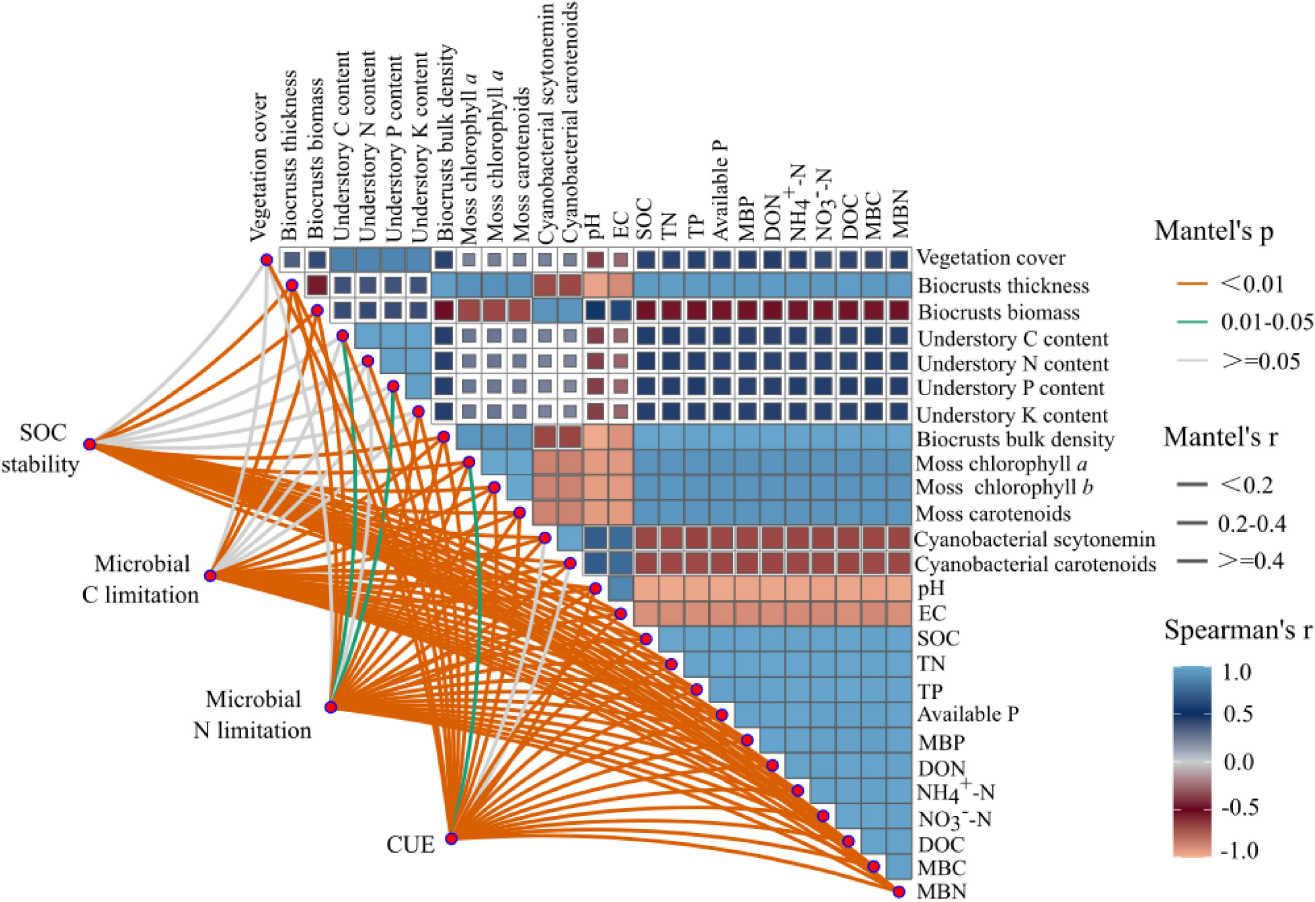
Mantel tests were employed to assess the correlations among understory vegetation, biocrust properties, photosynthetic pigments, soil nutrients, microbial biomass, soil organic carbon stability, microbial nutrient limitation, and microbial carbon use efficiency. The width of each edge is proportional to the Mantel’s r statistic, reflecting the strength of the correlation, while the color represents the statistical significance (*P*-value).

Mantel tests revealed that biocrust characteristics, photosynthetic pigments, and soil physicochemical properties significantly influenced organic carbon stability and microbial C and N limitation (*P* < 0.001; Figure 5). Furthermore, all examined factors significantly affected microbial CUE, with the exception of understory plant C, N, P, and K content and biocrust photosynthetic pigments.

**FIGURE 6:**
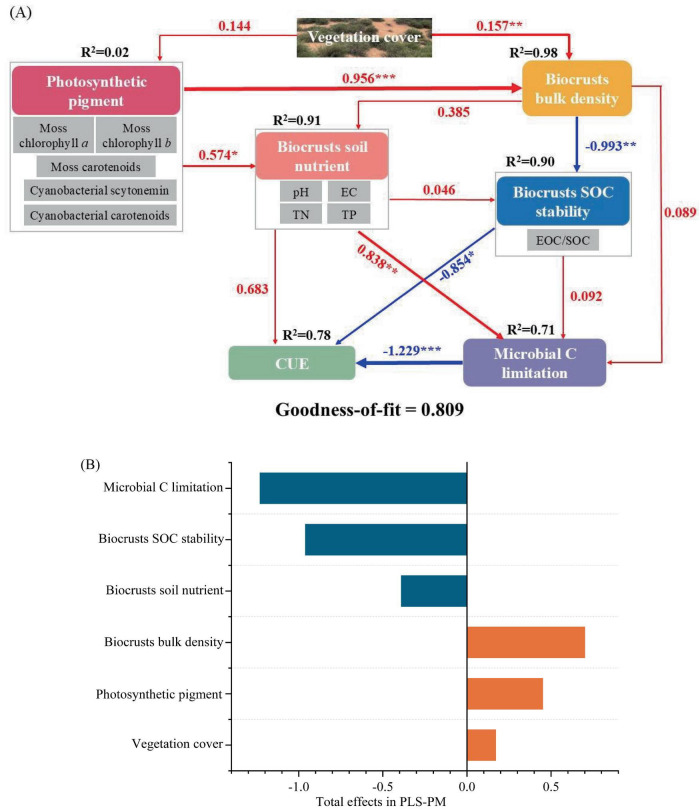
Partial least squares path modeling (PLS-PM) was applied to elucidate the principal pathways governing microbial carbon use efficiency in biocrust. The model illustrates the significant effect pathways in **(A)** and quantifies the total effects of various predictors on microbial carbon use efficiency in **(B)**. Significant (*p* < 0.05) positive and negative causal associations are marked by red and blue arrows, respectively, with the corresponding standardized path coefficients indicated. *R*^2^ values represent the proportion of variance explained for each dependent variable. Significance levels are denoted as follows: ***P < 0.001; **P < 0.01; *P < 0.05.

The PLS-PM results elucidated the direct and indirect relationships between vegetation cover, biocrust properties, and microbial metabolic functions (Figure 6). Vegetation cover directly affected photosynthetic pigments (path coefficient = 0.141) and bulk density (0.157). Photosynthetic pigments strongly increased bulk density (0.956) and nutrient content (0.574). Bulk density exhibited a strong negative direct effect on organic carbon stability (−0.993) and a positive effect on microbial C limitation (0.089). Furthermore, biocrust nutrients positively influenced microbial C limitation (0.838) and CUE (0.683). In contrast, organic carbon stability and microbial C limitation both exerted significant negative direct effects on CUE (−0.854 and −1.229, respectively). Overall, microbial C limitation had the strongest negative effect on CUE (Figure 6B).

## 4 Discussion

### 4.1 Drivers of biocrust patterns across mining disturbance gradients and vegetation types

A distinct ecological distribution pattern was observed for biocrusts in semi-arid mining subsidence areas. Consistent with our hypothesis, we found that coal mining disturbance altered the distribution of moss and cyanobacterial biocrusts. Specifically, moss cover was significantly higher in the herbaceous treatments than in the shrub treatments, whereas cyanobacterial cover was significantly higher in the shrub treatments than in the herbaceous treatments (Table 1). This distribution was likely driven by the contrasting microenvironments created by the two vegetation types. Shrubs typically provide incomplete ground cover, resulting in larger areas of bare soil that receive more solar radiation (Table 1). These conditions favor cyanobacteria, which can adapt their metabolism to recurring hydration-desiccation cycles or enter dormancy under drought (Rajeev et al., 2013), excrete exopolysaccharides to mitigate cellular moisture loss and stabilize soil particles (Rossi et al., 2018), and synthesize UV-screening pigments such as scytonemin (Garcia-Pichel and Castenholz, 1991). In contrast, herbaceous vegetation can effectively reduce near-ground wind speed and decrease water evaporation. Growing close to the ground creates a microclimate with higher humidity and a more stable temperature. This cool, humid environment is preferred by mosses (Ladrón, de Guevara and Maestre, 2022) and precisely meeting the requirements for widespread colonization.

Mining disturbance gradients significantly alter biocrust characteristics and photosynthetic pigments. Both cyanobacterial and moss biomass were highest in unmined areas, intermediate in 1–2 year mined-out areas, and lowest in active mining areas (Table 2). This may be attributed to adverse environmental changes induced by coal mining subsidence, including declining groundwater levels, loss of soil water and nutrients, and surface degradation (Zhang et al., 2023). Another possible explanation is that mining subsidence may affect biocrust distribution and characteristics by altering soil particle transport and water redistribution (Wu and Zhang, 2018; Lu et al., 2025). Biocrusts pigments are generally divided into two main categories—photosynthetic and protective pigments (Lan et al., 2012). Photosynthetic pigments absorb light energy to drive photosynthesis and supply energy and organic matter to ecosystem inhabited by biocrusts, thereby supporting their development and succession. Our results indicate that active mining significantly reduced the content of key photosynthetic pigments in biocrusts, including chl *a*, chl *b*, and carotenoids. Furthermore, our results indicated that the content of scytonemin, an essential photoprotective pigment in cyanobacteria, was significantly highest in unmined areas and lower in areas under active mining. This finding aligns with observed changes in cyanobacterial coloration—dark cyanobacteria predominated in unmined areas, whereas light cyanobacteria were more common in active-mining areas. These results suggest that mining disturbances degrade well-developed biocrusts originally, ultimately reversing the successional pathway from a mature, moss-dominated state to an earlier, cyanobacteria-dominated stage (Faist et al., 2017).

The pH of the cyanobacterial biocrusts was significantly higher than that of the moss biocrusts (Supplementary Table 1). This finding is consistent with previous reports of higher pH in cyanobacterial and lichen biocrusts than in moss biocrusts (Zhao H. L. et al., 2010; Atashpaz et al., 2023). The observed acidification under moss biocrusts likely results from the pronounced release of organic acids and respiratory carbon dioxide (Atashpaz et al., 2023), a mechanism that underscores the role of biocrust functional type in regulating soil microenvironments. Furthermore, biocrust development and succession drive pedogenic improvements, including increased clay and silt content that supports soil aggregate formation and enhances nutrient adsorption (Wang et al., 2025). This pedogenic function is particularly pronounced in moss biocrusts, which not only benefit from improved soil structure but also directly intercept atmospheric nutrients owing to low cell differentiation and a high density of cation exchange sites on their leaves (Sheng et al., 2023). These mechanisms collectively lead to a significant nutrient enrichment in moss biocrusts, as evidenced by their higher levels of TN, TP, AP, NO_3_^–^-N, NH_4_^+^-N, DON, MBN, and MBP than in cyanobacterial biocrusts across all studied vegetation and disturbance contexts (Supplementary Table 1), highlighting the role of mosses in ecosystem nutrient accumulation during succession.

### 4.2 Effects of mining disturbance gradients and vegetation types on organic carbon fractions and stability in biocrusts

The organic carbon content of biocrusts originates primarily from vascular plant litter and photosynthetic fixation (Aranibar et al., 2022; Zhang et al., 2024). Our results showed that active mining significantly reduced organic carbon across all biocrust-vegetation combinations compared with unmined areas in the same treatments, with decreases of 8.67% (moss in herbaceous), 11.37% (cyanobacterial in herbaceous), 7.25% (moss in shrub), and 12.87% (cyanobacterial in shrub) (Figure 2A). This reduction could be attributed to two factors. First, the herbaceous treatment of unmined areas had significantly greater vegetation and litter cover, higher understory nutrient concentrations (C, N, P, K), and greater topsoil root biomass (Table 1; *P* < 0.05; Li et al., 2023), all of which are critical sources of organic matter for biocrusts (Zhang et al., 2024). Second, mining activities have induced a pronounced decline in soil clay and silt fractions, with reductions of up to 50% in their content (Ma et al., 2019). This degradation of soil texture leads to poorer soil physicochemical properties, which, in turn, inhibit plant growth and further limit organic matter inputs (Li et al., 2022). DOC and MBC are primarily derived from microbial decomposition of organic matter, and their concentrations are largely determined by the extent of microbial degradation (Huang et al., 2022). EOC mainly originates from the decomposition of plant waste, root secretions, and the degradation of organic matter driven by soil microorganisms (Sun et al., 2023). Our findings revealed that DOC, MBC, and EOC contents in biocrusts were significantly lower in actively mined and 1–2-year mined-out areas than in unmined areas. This reduction can be explained by land subsidence caused by underground mining (He et al., 2020), which modifies soil physicochemical properties (Wang et al., 2019), disrupts the microbial community structure (Huang et al., 2020), and consequently impairs the stable physical substrate and microenvironment essential for biocrust survival and carbon sequestration. The ROC primarily originates from root inputs and represents a stable fraction of the SOC pool (Lavallee et al., 2020). Our results demonstrated that herbaceous treatment significantly increased ROC content in moss biocrusts, whereas shrub treatment led to a pronounced increase in ROC content in cyanobacterial biocrusts. These findings suggest that herbaceous and shrub vegetation contribute to the stabilization of the organic carbon pool in moss and cyanobacterial biocrusts, likely attributable to distinct microenvironmental conditions and root-mediated carbon inputs associated with each vegetation type.

Elevated EOC/SOC and EOC/ROC ratios indicate lower stability of the SOC pool, attributable to the relatively rapid turnover and greater vulnerability of these fractions to oxidative degradation (Thaysen et al., 2017). Conversely, an increased ROC/SOC ratio indicates a more recalcitrant SOC composition that is less amenable to decomposition. In this study, cyanobacterial biocrusts showed significantly higher EOC/SOC and EOC/ROC ratios (increases of 9.54–15.27 and 143.76–177.14%, respectively) than moss biocrusts. In comparison, the ROC/SOC ratio was substantially greater in the moss biocrusts, exceeding that in the cyanobacterial biocrusts by 118.48–142.67% (Table 3). Collectively, these results suggest that moss biocrusts possess markedly higher organic carbon stability than cyanobacterial biocrusts. This divergence may be explained by differences in the biochemical exudates produced during biocrust development. Previous studies have established that cyanobacteria excrete polysaccharides that promote the cementation of filaments with soil particles and co-occurring organisms (Belnap et al., 2016). Meanwhile, moss biocrusts at advanced successional stages accumulate phenolic compounds structurally analogous to lignin, which confer greater resistance to microbial breakdown owing to their complex molecular configurations (Yang et al., 2019).

### 4.3 Effects of mining disturbance gradients and vegetation types on microbial metabolism in biocrusts

Soil extracellular enzymes are pivotal mediators of key biochemical processes, including organic matter decomposition, nutrient cycling, and energy flow, and are recognized as sensitive indicators of ecosystem functioning (Dove et al., 2020). These enzymes primarily originate from microorganisms, plant roots, and soil fauna, and their activities are modulated by various biotic and abiotic factors, including moisture, temperature, and nutrient availability (Ghiloufi et al., 2019). Our study revealed that the extracellular enzyme activity was significantly higher in moss than in cyanobacterial biocrusts (Supplementary Figure 1). This may be attributed to the greater biomass and higher nutrient levels associated with moss biocrusts, which provide ample carbon and nutrients to soil microorganisms, thereby fostering conditions conducive to increased microbial activity. This improves soil respiration and enzyme production (Liu et al., 2017). Additionally, moss biocrusts exhibit a stronger capacity to regulate soil moisture and temperature (Guan et al., 2020), thereby mitigating environmental stress, a critical determinant of enzyme activities in soil biogeochemical cycling. In arid and semi-arid regions, water availability is the primary limiting factor for ecosystem productivity and a key driver of soil enzyme activity involved in C, N, and P biogeochemical cycling (McDaniel et al., 2013; Kakeh et al., 2018). Our findings indicated that the activities of C-, N-, and P-acquiring enzymes were highest in unmined areas, followed by 1–2-year mined-out areas, with active mining areas exhibiting the lowest levels (Supplementary Figure 1). This trend may be attributed to greater vegetation and litter cover in the unmined areas, which reduced evaporation of soil moisture. Improved moisture conditions, coupled with organic inputs, have been shown to alter microbial composition and enhance metabolic activity in biocrust systems (Geisseler et al., 2011).

As a stable element derived primarily from the parent rock, phosphorus in soil systems is largely accessed by microorganisms via the breakdown of organic matter (Razavi et al., 2016). Vector angle analysis revealed phosphorus limitation (angle > 45°) in moss biocrusts from both undisturbed and 1–2 year mined-out areas under herbaceous and shrub treatments, with a significantly stronger limitation observed in recently disturbed areas. Concurrently, vector length, which denotes microbial carbon limitation, was markedly higher in moss biocrusts than in cyanobacterial biocrusts (Figure 3). This enhanced C and P co-limitation likely results from two interacting factors—the inherently low phosphorus content of parent material in mining subsidence areas and the dense physical structure of moss biocrusts, which, while conserving soil moisture, also reduces the atmospheric input of organic particles (e.g., aeolian dust and plant debris), thereby constraining external carbon flux into the biocrust system. Cyanobacteria are widely regarded as key contributors to soil nitrogen fixation during biocrust succession processes (Nawaz et al., 2024). Contrary to expectations, our findings revealed that cyanobacterial biocrusts exhibited stronger nitrogen limitation (vector angles < 45°), which was inconsistent with the established understanding. This discrepancy may be attributed to the semi-arid to arid climatic conditions of the study region, in which limited precipitation constrains the nitrogen-fixation capacity of the cyanobacterial biocrust (Zhao Y. G. et al., 2010). Thus, moisture scarcity is likely the primary factor driving the pronounced nitrogen limitation in these biocrust systems.

### 4.4 Key factors driving organic carbon stability and microbial metabolic characteristics in biocrusts

SOC stability is regulated by a combination of environmental drivers (e.g., soil temperature, moisture, and rainfall patterns) (Yao et al., 2020), soil texture (Yu et al., 2019), and vegetation type (e.g., forests, shrubs, and grasses) (Gao et al., 2020). Previous studies have indicated that SOC accrual and stabilization in biocrust profiles result from the interplay between plant inputs, soil physicochemical traits, microbial community structure, carbon-degrading enzyme activity, and MBC (Li et al., 2025). Consistent with these mechanisms, our results further demonstrated that biocrust characteristics (e.g., thickness and biomass), photosynthetic pigments (e.g., chl *a* and *b*), and soil physicochemical parameters (e.g., MBC, MBN, and MBP) significantly affected organic carbon stability (*P* < 0.001); (Figure 5). These results enhance our understanding of carbon dynamics in arid-land biocrusts and highlight the importance of promoting biocrust development for sustained SOC storage.

The PLS-PM analysis demonstrated a significant negative direct effect of microbial carbon limitation on CUE (Figure 6), consistent with earlier studies (Cui et al., 2020; Du et al., 2025). This effect was primarily due to the role of soil nutrient availability in microbial metabolic functions. Enhanced carbon limitation induces a transition in the soil microbial metabolic strategy from growth to maintenance of respiration, accompanied by elevated extracellular enzyme synthesis and secretion, collectively reducing CUE (Yu et al., 2022). Additionally, microbial carbon limitation imposes a strong negative regulatory effect on CUE by disturbing the balance between microbial respiration and biomass production (He et al., 2023). Microbial CUE is a key indicator of the balance between carbon allocated to biomass production and respiration during microbial metabolism (He et al., 2024). Our results revealed a significant direct negative effect of organic carbon stability on CUE (Figure 6). This relationship could be attributed to two primary mechanisms. First, stable organic carbon is predominantly composed of complex compounds such as humic substances and physically protected carbon fractions, which are characterized by high molecular weight and intricate chemical structures, thus limiting their direct assimilation by microorganisms (Martinović et al., 2022). Secondly, when exposed to recalcitrant substrates, microorganisms activate energy-intensive catabolic pathways, thereby increasing respiratory flux to meet the energy demands of cellular maintenance and extracellular enzyme synthesis (Manzoni et al., 2012). Consequently, the proportion of carbon allocated to respiration increased significantly, whereas that allocated to growth decreased, thereby causing a marked reduction in microbial CUE.

## 5 Conclusion

(1) Coal mining subsidence, particularly in active mining areas, may reduce biocrust cover, nutrient availability, organic carbon stability, microbial enzyme activities involved in C, N, and P acquisition, and microbial CUE, confirming coal mining as an important factor affecting the stability of organic carbon and microbial nutrients limitation in biocrusts.

(2) The effects of mining disturbance gradients and vegetation types on microbial CUE in biocrusts were attributed to the combined influence of multiple factors, including vegetation cover, photosynthetic pigments content, physicochemical properties, and biocrust bulk density.

(3) Coal mining disturbance subsidence profoundly affects the stability of biocrust carbon and microbial metabolic functioning. Microbial carbon limitation and organic carbon stability emerged as key regulators of microbial CUE, providing mechanistic insights into biocrust-mediated carbon cycling and a theoretical basis for the ecological restoration and sustainable management of coal mining subsidence ecosystems.

## Data Availability

The original contributions presented in this study are included in this article/Supplementary material, further inquiries can be directed to the corresponding author.
