# Supplementary material for: Underground coal mining reduces organic carbon stability and alters microbial metabolic limitation in biocrusts of an arid region in northern China
Source: Front Microbiol. 2026 Jun 23;17:1798269. doi: 10.3389/fmicb.2026.1798269 (PMC13338762; doi:10.3389/fmicb.2026.1798269)
Supplement: Supplementary file 1 [file Supplementary_file_1.pdf]

Underground coal mining reduces organic carbon stability and alters microbial metabolic limitation in biocrusts of an arid region in northern China

Yun Guo [a*], ShaoPeng Ma [b,c], Li Ma [a], Benhua Sun [d], ManYin Zhang [a]

[a] *Institute of Geological Hazards Prevention, Gansu Academy of Sciences, Lanzhou 730000, China*

[b] *Institute of Geographical Sciences, Hebei Academy of Sciences, Hebei Technology Innovation Center for Geographic Information Application, Shijiazhuang 050011, China*

[c] *Postdoctoral Scientific Research Station of Geography, Hebei Normal University, Shijiazhuang 050024, China*

[d] *Key Laboratory of Plant Nutrition and the Agri-Environment in Northwest China, Ministry of Agriculture, Northwest A&F University, Yangling 712100, People's Republic of China*

* Corresponding author: Yun Guo

E-mail address: xnguoyun@163.com

Supplementary material

Table S1 Effects of mining disturbance gradients on biocrust soil properties

Biocrust soil properties	Unmined area				Active mining area				1-2 Year mined-out area			
	Herbaceous		Shrub		Herbaceous		Shrub		Herbaceous		Shrub	
	Moss	Cyano	Moss	Cyano	Moss	Cyano	Moss	Cyano	Moss	Cyano	Moss	Cyano
pH	7.53 ± 0.01^{l}	7.90 ± 0.01^{c}	7.56 ± 0.01^{k}	7.86 ± 0.01^{f}	7.64 ± 0.01^{h}	8.01 ± 0.01^{a}	7.67 ± 0.01^{g}	8.00 ± 0.01^{b}	7.61 ± 0.01^{j}	7.89 ± 0.01^{d}	7.62 ± 0.01^{i}	7.88 ± 0.01^{e}
EC (μs·cm^{-1})	46.7 ± 0.4^{j}	58.6 ± 0.5^{e}	47.9 ± 0.3^{i}	60.8 ± 0.7^{d}	50.9 ± 0.3^{g}	65.6 ± 0.7^{a}	52.2 ± 0.4^{f}	57.9 ± 0.2^{e}	48.7 ± 0.4^{h}	62.0 ± 0.4^{c}	51.1 ± 0.3^{g}	63.8 ± 0.8^{b}
TN (g·kg^{-1})	1.58 ± 0.01^{a}	0.83 ± 0.01^{f}	1.50 ± 0.01^{b}	0.91 ± 0.01^{e}	1.16 ± 001^{d}	0.70 ± 0.01^{h}	1.16 ± 0.01^{d}	0.70 ± 0.01^{h}	1.33 ± 0.01^{c}	0.78 ± 0.01^{g}	1.33 ± 0.01^{c}	0.79 ± 0.01^{g}
TP (g·kg^{-1})	0.57 ± 0.01^{a}	0.26 ± 0.01^{f}	0.51 ± 0.01^{b}	0.28 ± 0.01^{e}	0.37 ± 0.01^{d}	0.17 ± 0.01^{h}	0.37 ± 0.01^{d}	0.18 ± 0.01^{h}	0.45 ± 0.01^{c}	0.24 ± 0.01^{g}	0.45 ± 0.01^{c}	0.24 ± 0.01^{g}
AP (mg·kg^{-1})	24.7 ± 0.01^{a}	14.8 ± 0.14^{f}	24.2 ± 0.07^{b}	15.5 ± 0.12^{e}	20.0 ± 0.05^{d}	12.3 ± 0.07^{h}	20.0 ± 0.01^{d}	12.4 ± 0.02^{h}	22.5 ± 0.22^{c}	12.6 ± 0.02^{g}	22.4 ± 0.21^{c}	12.6 ± 0.07^{g}
NO_3^--N (mg·kg^{-1})	17.9 ± 0.03^{a}	12.0 ± 0.15^{f}	17.3 ± 0.07^{b}	12.1 ± 0.05^{e}	12.3 ± 0.02^{d}	7.6 ± 0.03^{h}	12.3 ± 0.06^{d}	7.6 ± 0.03^{h}	15.3 ± 0.09^{c}	10.4 ± 0.05^{g}	15.3 ± 0.05^{c}	10.4 ± 0.02^{g}
NH_4^+-N (mg·kg^{-1})	10.4 ± 0.05^{a}	8.0 ± 0.02^{f}	9.9 ± 0.06^{b}	8.2 ± 0.03^{e}	8.4 ± 0.08^{d}	5.6 ± 0.04^{h}	8.4 ± 0.08^{d}	5.6 ± 0.03^{h}	9.5 ± 0.04^{c}	7.3 ± 0.03^{g}	9.4 ± 0.04^{c}	7.3 ± 0.03^{g}
DON (mg·kg^{-1})	64.9 ± 0.08^{a}	48.1 ± 0.04^{f}	63.4 ± 0.07^{b}	50.4 ± 0.11^{e}	58.5 ± 0.06^{d}	43.1 ± 0.02^{h}	58.4 ± 0.11^{d}	43.1 ± 0.02^{h}	62.7 ± 0.08^{c}	46.7 ± 0.07^{g}	62.6 ± 0.12^{c}	46.8 ± 0.04^{g}
MBN (mg·kg^{-1})	105.3 ± 0.14^{a}	77.0 ± 0.01^{f}	95.2 ± 0.04^{b}	82.2 ± 0.10^{e}	87.2 ± 0.32^{d}	64.1 ± 0.30^{h}	86.7 ± 0.76^{d}	64.5 ± 0.24^{h}	92.0 ± 0.07^{c}	75.1 ± 0.07^{g}	91.8 ± 0.27^{c}	75.1 ± 0.10^{g}
MBP (mg·kg^{-1})	13.6 ± 0.03^{a}	7.7 ± 0.04^{f}	12.2 ± 0.12^{b}	7.9 ± 0.04^{e}	11.5 ± 0.04^{d}	6.2 ± 0.04^{h}	11.5 ± 0.10^{d}	6.3 ± 0.04^{h}	11.9 ± 0.07^{c}	6.6 ± 0.06^{g}	11.9 ± 0.10^{c}	6.6 ± 0.02^{g}

Significant differences ($P < 0.05$) within a row are represented by different lowercase letters, based on Duncan's multiple range test. Data are mean ± standard deviation. Cyano, Cyanobacterial; EC, electrical conductivity; TN, total nitrogen; TP, total phosphorus; AP, available phosphorus; NO_3^--N, nitrate nitrogen; NH_4^+-N, ammonium nitrogen; DON, dissolved organic nitrogen; MBN, microbial biomass nitrogen; MBP, microbial biomass phosphorus.

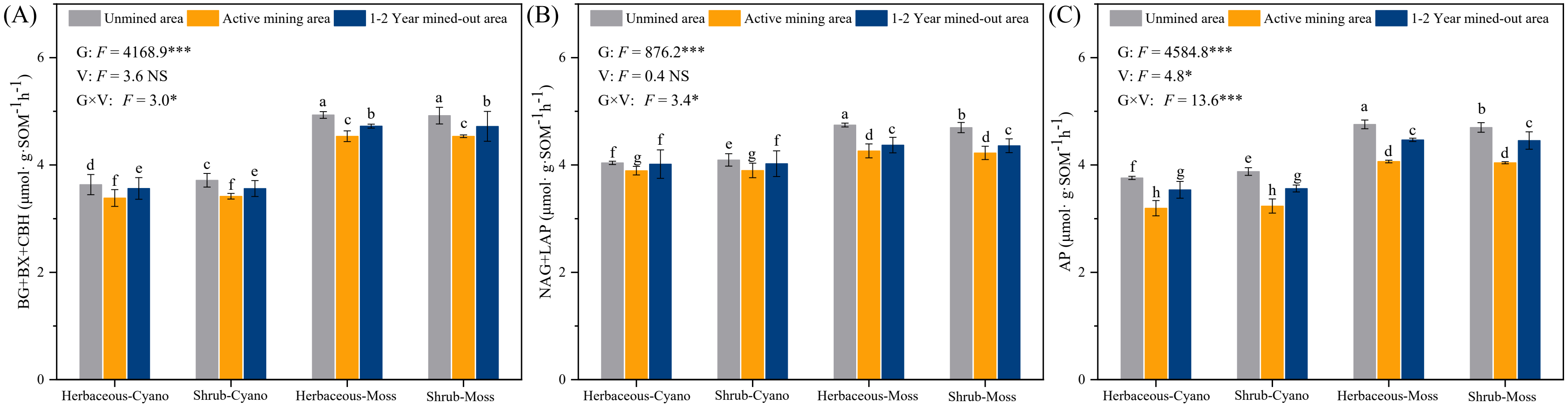

Fig. S1 Effects of mining disturbance gradients on biocrust soil extracellular enzyme activities. (A) carbon-related extracellular enzyme activities, (B) nitrogen-related extracellular enzyme activities, (C) phosphorus-related extracellular enzyme activities. BG, β-1,4-glucosidase; BX: β-1,4-xylosidase; CBH, β-D-cellobiosidase; NAG, β-1,4-N-acetylglucosaminidase; LAP, L-leucine aminopeptidase; AP, acid phosphatase. Data are mean ± standard deviation. Bars with different letters indicate significant differences at $P < 0.05$. The abbreviations "G", "V," and "G × V" indicate individual and interaction effects of different gradients of mining disturbance and the two vegetation types. ***$P < 0.001$; *$P < 0.05$; NS no significant difference.
